# Doctors’ Perceptions of E-Prescribing upon Its Mandatory Adoption in Poland, Using the *Unified Theory of Acceptance and Use of Technology* Method

**DOI:** 10.3390/healthcare8040563

**Published:** 2020-12-15

**Authors:** Natalia Wrzosek, Agnieszka Zimmermann, Łukasz Balwicki

**Affiliations:** 1 Department of Medical and Pharmacy Law, Medical University of Gdańsk, 80-210 Gdańsk, Poland; natalia.wrzosek@gumed.edu.pl; 2 Department of Public Health & Social Medicine, Medical University of Gdańsk, 80-210 Gdańsk, Poland; balwicki@gumed.edu.pl

**Keywords:** electronic prescribing, UTAUT, attitude to computers

## Abstract

Background: E-prescribing is the most important achievement in the automation of the healthcare sector in Poland. Obligatory electronic prescribing came into force on 8 January 2020. This innovation significantly changes the work of doctors. Therefore, it is useful to identify the factors that have an impact on doctors’ acceptance levels for the new technology. Methods: This study employs a survey based on the Unified Theory of Acceptance and Use of Technology (UTAUT) method. Our questionnaire was completed by 144 family doctors in Poland during the technology implementation phase and the initial period of obligatory e-prescribing, between 1 December 2019 and 1 March 2020. Results: The results of the survey indicate that doctors do not believe that e-prescribing improves the effectiveness of their work. In addition, this attitude does not depend on the age of the respondent. We also found, regarding the influence of societal attitudes, that doctors only rarely consider the opinions of others in relation to their job. Conclusions: The implementation of new technologies in the healthcare system must be accompanied by consideration of how user-friendly the technologies are, and whether the users will have appropriate technical and financial support.

## 1. Introduction

eHealth refers to the use of Information and Communication Technologies (ICT) in health products, services and processes, combined with organizational changes in healthcare systems; new skills, to improve health outcomes and the efficiency and productivity of healthcare delivery and the economic and social value of health. eHealth covers the interaction between patients and health-service providers, the institution-to-institution transmission of data and peer-to-peer communication between patients and/or health professionals [1]. Using ICT leads to the transition from healthcare to eHealth. Today, a wide range of technologies like apps, telemedicine, electronic medical records, ‘connected medicine’ and ‘smart homes’ are in everyday use.

In the European Union, under the “eEurope 2005 Information Society for All” protocol [2], member states are obliged to develop e-services, particularly in the healthcare sector [3]. The first detailed guidelines of the European Commission of 2007 concerned, among others, the introduction of electronic medical services, like e-prescribing, e-referrals, telecare and teleadvice [4]. Adopting the Directive on the application of patients’ rights in cross-border healthcare in 2011 was another step towards international cooperation in the field of eHealth. Article of 14 of the Directive focuses on regulations connected with the implementation of eHealth services across Europe in order to maximise social and economic benefits [5]. In the paper entitled “eHealth Action Plan 2012–2020: Innovative healthcare for the 21st century”, the European Commission pointed out, however, that, despite some significant progress, there were still barriers to a fully mature and interoperable eHealth system [1].

In Poland, eHealth implementation commenced in 2008 with the approval of Government Resolution No 274/2008 in relation to the document titled “Information Society Development Strategy in Poland till 2013” [6].

eHealth priorities include, without limitation:

- improving the effectiveness of the healthcare system with regard to the circulation of electronic medical documentation;
- analysing a demand for healthcare services through the modernisation of a medical information system; and
- developing IT healthcare solutions which will be consistent with the European Commission’s guidelines and will enable Poland’s accession to the interoperable Electronic Health Record (EHR) area [7].

E-prescribing is a central ICT system made available under the “Electronic Platform for Collecting, Analysing and Sharing Digital Resources Related to Medical Events” (hereinafter referred to as the P1 System). This is one of key tools implemented during the automation of the healthcare system. In Poland, there was an initial pilot testing stage, where e-prescribing was tested in 19 entities, and then, after certain changes were made to the system, and with many more healthcare centres expressing their desire to take part, the system of e-prescribing was expanded across the whole country. The pilot stage officially launched on 25 May 2018 in the city of Siedlce (central Poland). Data from the pilot project enabled a determination of which elements of the e-prescribing system were operating effectively and which needed improvement. During the pilot project, the Government held meetings with project participants, which enabled lessons to be learned first-hand from those implementing the system. The most significant problems were those associated with the diversity of e-prescribing software applications on the market for pharmacies and healthcare centres to use [8]. This problem had not been resolved at the date of writing.

From 1 January 2019, medicines issued by e-prescription were available in every pharmacy in Poland. Healthcare centres, such as clinics, hospitals and private doctors, were required to connect to the P1 System by 31 December 2019 at the latest. On 8 January 2020, obligatory e-prescribing was implemented across Poland [9]. The legislation provided for a limited set of circumstances where paper prescriptions might still be issued. For instance, a paper prescription could be issued where there was no access to the P1 system; or where the person to whom the prescription is being issued cannot be identified (e.g., an unconscious or homeless person); or where the prescribing nurse or doctor is whose professional certification has been issued in another EU member state and who is working temporarily in Poland; or for a medicinal products that do not have marketing authorisation in Poland and are imported from abroad authorized by direct import regulations [10].

Overseas studies have shown that e-prescribing brings many benefits. One intended benefit was to reduce the number of prescription errors especially those resulting from the illegibility of handwritten prescriptions [11,12]. A 2016 literature review of 73 scientific publications on the advantages and risks connected with the implementation of e-prescribing, estimated that 83% of all studies indicated that the number of medical errors had been reduced upon the implementation of electronic prescribing, 81% of publications confirmed related cost reductions for the healthcare system, and 76% of studies showed that e-prescribing had increased the quality of healthcare services and improved patient safety [13].

The past 25 years has seen new technologies enter all aspects of people’s lives. Producers have also been compelled to conduct thorough research on consumer likings and preferences, and over the years there have been many research models developed to analyse the use of new technologies. The first models were developed in 1985 and have been systematically modified to suit changing systems and markets for new technologies [14]. Acceptance theories and models developed over the years, e.g., Technology Acceptance Model (TAM), Unified Theory of Acceptance and Use of Technology (UTAUT) or D&M Information Systems Success Model (D&M IS Success)*,* have become important analytical tools for software and hardware producers in many industries. The aforementioned research models can be flexibly modified and adjusted to various research scenarios [15]. These models enable researchers to assess the impact of factors such as usability, ease of use, anticipated difficulties, influences on consumer conduct and consumer acceptance for any given technology.

The outbreak of the COVID-19 pandemic in late 2019, and its continuation throughout 2020, have contributed to an increased interest in remote work, online education, socially distanced care for the elderly [15]. Healthcare systems have also increased their use of computer tools to a much greater extent.

The efficient and effective implement of ICT innovations in the healthcare sector depends on learning and understanding the factors that have an impact on medical professionals’ attitudes and approaches to the new technologies. It is important to identify those features and functionalities of the tools that, in doctors’ opinions, are essential to make their everyday work more efficient and effective [16]. Within the ICT sector, surveys of the acceptance of new technologies, using a variety of research methods, are highly developed. However, despite the available of suitable research tools, there are few studies of these factors applied in the healthcare system [17]. Therefore, the purpose of our survey was to identify those aspects of the e-prescription system in Poland that best facilitated doctors’ willingness to use the e-prescribing system during the system’s implementation stage. Additionally, this study sought to verify the hypotheses that age, gender or level of professional experience affect the perception of e-prescription, especially the level of expected performance or anticipated implementation difficulties.

### Conceptual Framework

The present study was based on the Unified Theory of Acceptance and Use of Technology (UTAUT) model, which is considered to be the model best suited to studying new technology users’ motivation and conduct [18]. Considering the situation in Poland, our survey applied the UTAUT model to identify the factors that influenced doctors’ acceptance and use of the electronic prescribing system. The UTAUT methodology considers four major determinants of the use and acceptance of new technologies:

(a) Performance expectancy (PE): a degree of belief that the use of a given technology improves the performance of important duties.
(b) Effort expectancy (EE): the motivational impact of the expected effort required to use a technology. The expected effort decreases over time with ongoing use of the technology, as increasing experience in the use of the technology has a positive impact on the users’ willingness to use the technology.
(c) Social influence (SI): a degree of belief that other people would also use a technology. This variable has a greater impact on a user’s intention to use the technology at the early stage of using of the technology and decreases with time as the user gains experience.
(d) Facilitating conditions (FC): the degree to which a given person is convinced that there is the relevant technical and organisational infrastructure to provide support in case there are difficulties experienced when using the technology [19].

In addition, the UTAUT takes into account the independent variables of gender, age and experience of users [20]. It also aggregates the influence of performance expectancy, effort expectancy and social influence into the variable ‘behavioural intentions.’ Facilitating conditions directly affect “use behaviour”. These factors are treated as dependent variables within the model. Some modifications of the model also consider whether there is voluntary of use of the technology and the frequency of its use. Internationally, this methodology has been applied many times in surveys conducted in the healthcare sector [17,21,22,23], including in the assessment of users’ acceptance of e-prescribing [16]. The details of each answer to the questions were also been assessed for the presence of any significant differences within each section separately.

## 2. Materials and Methods

### 2.1. Study Design

**Figure 1:**
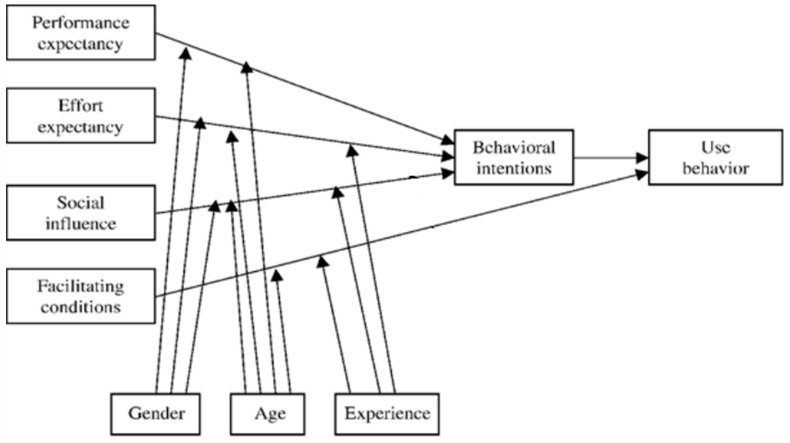
Unified Theory of Acceptance and Use of Technology (UTAUT) model used in the survey [18] with modifications matching the specifics of the Polish healthcare system (i.e., e-prescribing is mandatory in Poland, not voluntary)**.**

Our study’s analysis of the pilot e-prescribing implementation project data was based on the model presented in Figure 1.

Each respondent’s gender, age and experience were considered as external determinants likely to influence their response. [21]

**Figure 2:**
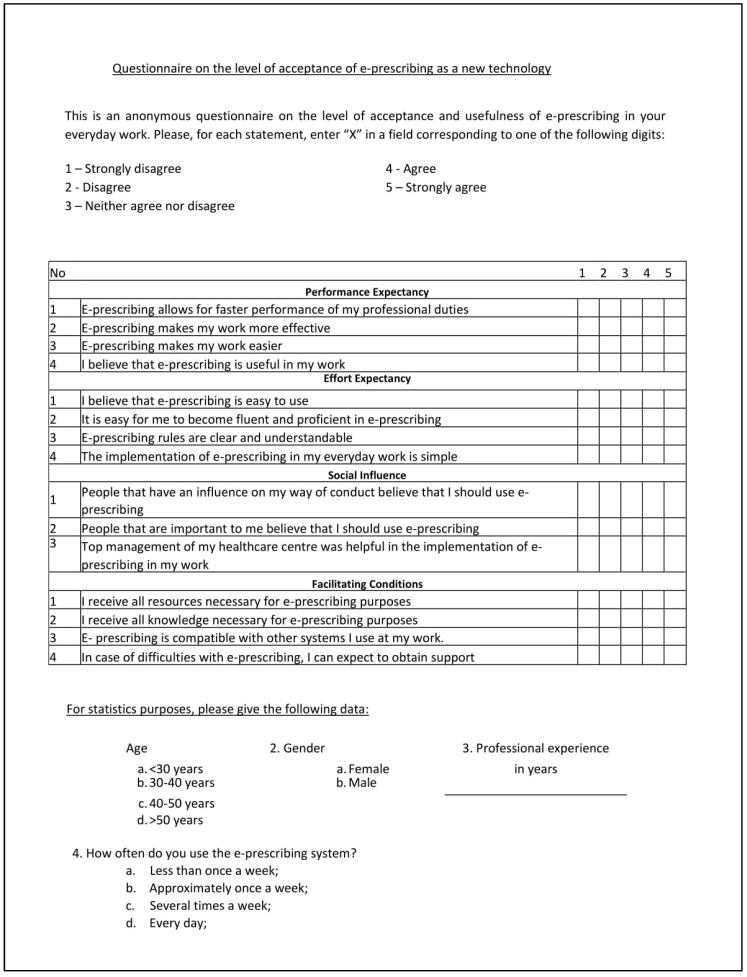
Original questionnaire used in the survey.

The questionnaire was prepared with the assumptions of the UTAUT model in mind (Figure 1). The questionnaire is a validated research tool. The questions were those been developed and adapted for the UTAUT model and have been standardised over time, enabling researchers to apply the questionnaire in many settings. Thus, in each question, a researcher may insert their own technology subject, and in our case, the term ‘e-prescription’ (Figure 2) [18].

### 2.2. Data Collection

The questionnaire was distributed by the researchers to private sector primary care doctors across Poland. Doctors taking part in the survey could either complete a paper-based questionnaire (distributed by one of the authors) or an electronic questionnaire (distributed by the researchers), based on their stated preference. The survey was addressed to currently active primary care doctors of various specialisations. The participants were informed that their responses were to be provided anonymously. Permission to conduct the survey at each healthcare centre was negotiated with centre managers on a case-by-case basis. The electronic form of the questionnaire was distributed via Google Forms and the associated information about the research project was provided to doctors in leaflet form. The completed questionnaires were collected between 1 December 2019 and 1 March 2020.

Polish Statistical Office data indicate that in 2018 there were 43,130 primary care doctors in Poland, who worked in 6711 healthcare centres. We calculated the representative sample size using an online calculator on www.raosoft.com/samplesize, with the result that a representative sample of a study population of 43,130 should include 381 respondents. The sample size was determined for a confidence level of 95% with a ±5% margin of error. In total, 400 questionnaires were distributed, with a return rate of 36%.

### 2.3. Ethical Considerations 

The Independent Bioethics Committee for Scientific Research at the Medical University in Gdańsk issued a positive opinion, approving on the survey as part of a larger research project entitled “The assessment of the usefulness of e-prescribing in the Polish healthcare system” (letter No. NKBBN/664/2018). The Committee assessed the project as a non-invasive research.

## 3. Results

The questionnaire was completed by 144 respondents, comprising 79 women and 64 men. Table 1 shows the age brackets of doctors taking part in the survey.

Most respondents were between the ages of 30 and 40 (41.26%). Before conducting further analysis, age was aggregated.

**Figure 3:**
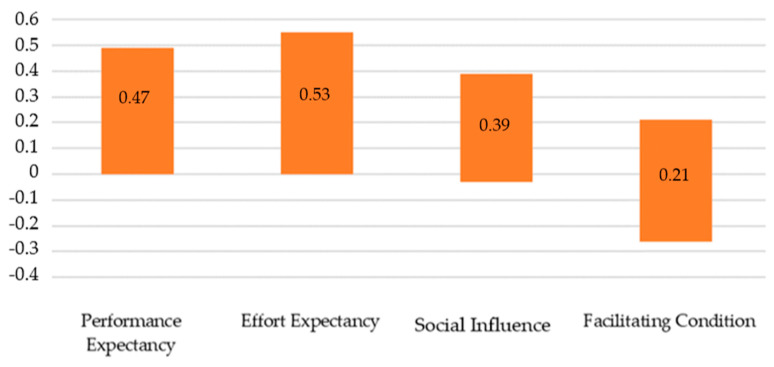
Confidence intervals B for individual predictors.

To compare the impact of each of the various factors identified in the UTAUT model on e-prescribing frequency, multiple linear regression was analysed in accordance with the input method. The analysis indicated that the suggested model with UTAUT factors as predictors was well-adjusted to data, F(4, 124) = 8.38; *p* < 0.001 and explained 21% of dependent variable variance, R^2^ = 0.21 (Table 2). Important predictors of e-prescribing frequency turned out to be the following areas: PE (β = 0.21; *p* < 0.05), EE (β = 0.23; *p* < 0.05) and SI (β = 0.16; *p* < 0.1), and SI was important in terms of a statistical tendency (Table 3). All the factors reflected a positive relation to e-prescribing frequency. The confidence intervals analysis identified no significant differences in the impact of those predictors on the acceptance of e-prescribing. The comparison of the confidence intervals for individual predictors in the model is presented on the graph. If these intervals overlap, it means that their strength does not differ from each other (Figure 3). Moreover, basic data analysis on the reliability of individual scales of the UTAUT questionnaire showed that internal consistency is satisfactory for all scales, except for Social Influence (Table 2).

In our analysis of responses given under the “Performance Expectancy” section, all statements were graded at an average of 2.06 using the five-point Likert scale.

**Figure 4:**
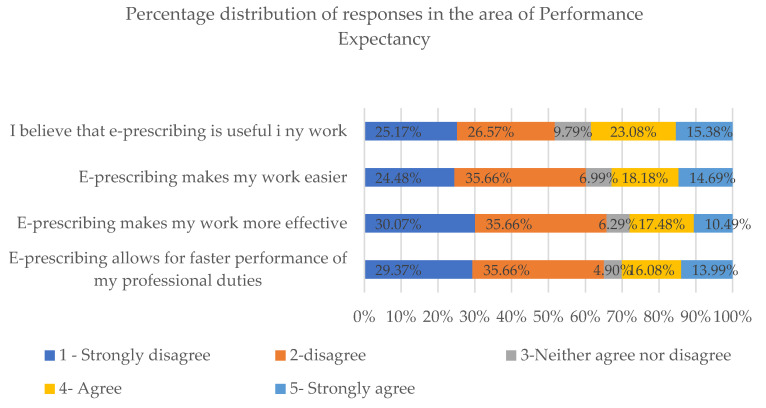
Percentage distribution of responses to questions in the Performance Expectancy section.

As regards questions in the Performance Expectancy section, most respondents answered “strongly disagree” or “disagree”. A trend of low responses is particularly noticeable in the case of questions concerning the impact of e-prescribing on the performance speed (65.03% of negative responses) and effectiveness (65.73% of negative responses) (Figure 4).

To verify our hypothesis that the age of the user would play a part in differentiating the impact of individual factors in the UTAUT model, a one-factor variance analysis in the intergroup scheme was conducted (Table 4). Age responses were an ordinal rather than a quantitative variable; therefore, analysis of variance was used.

Our initial analysis established that the results on the Effort Expectancy scale were differentiated according to age, F (2, 140) = 3.28; *p* < 0.05. However, post hoc analysis with the Bonferroni correction revealed no significant differences between the age groups in the Effort Expectancy scale results. Similarly, in the results from the other scales of the UTAUT Questionnaire did not show that age was a significant differentiating determinant.

In the Effort Expectancy area, the five-point Likert scale average was 3.13. However, it cannot be explicitly stated that the implementation of the new technology in doctors’ everyday work entails no problems.

It was identified that there was an important relation between professional experience and social influence on general acceptance of e-prescribing—the model was well adjusted to data, F (1, 141) = 4.18; *p* < 0.05 and explained 3% of dependent variable variance, R^2^ = 0.03. The longer professional experience, the higher responses in terms of Social Influence, β = 0.17; *p* < 0.05.

An average response in the five-point Likert scale in terms of Social Influence was 2.3. This area indicates to which extent respondents take the opinion of other people into account while planning their job. If each of the statements is analysed separately, it can be noticed that close people, like family members, have a definitely smaller impact on doctors’ professional approach (average: 2.89) than other “important people” that are perceived as an authority (average: 3.35). The same data indicate that most doctors did not feel sufficient support from their healthcare centre managers during the implementation of e-prescribing. In the scale from 1 to 5, this aspect was assessed at 2.97.

The results also confirmed with answers given in the Facilitating Conditions area. Most respondents confirmed that they disagreed or strongly disagreed that they could expect support in the case of difficulties connected with the operation of the e-prescribing system. 63.19% of the respondents gave a negative response to that question.

To verify the hypothesis that there is a material relation between professional experience and the level of UTAUT factors, a series of linear regression analyses were made.

As a result of the analysis, an important relation between professional experience and responses given in the Performance Expectancy section was identified—the model was well adjusted to data, F (1, 141) = 3.96; *p* < 0.05 and explained 3% of dependent variable variance, R^2^ = 0.03. The longer professional experience, the lower responses in this area, β = −0.17; *p* < 0.05. It was also identified that there was an important relation between professional experience and the level of effort perception—the model was well adjusted to data, F(1, 141) = 3.14; *p* < 0.1 and explained 2% of dependent variable variance, R^2^ = 0.02. The longer professional experience, the lower responses in this area, β = −0.15; *p* < 0.1. The data let us conclude that doctors with longer professional experience find e-prescribing as a tool that will facilitate their everyday work to a smaller extent and come across more difficulties during the implementation of the technology.

In the case of Facilitating Conditions, no material relation with professional experience was identified (Table 5).

The series of analyses conducted with the use of a *t*-Student test did not confirm the hypothesis that the gender has a material impact on the perception and acceptance of e-prescribing.

## 4. Discussion

Not all those authorised to issue prescriptions are enthusiastic about e-prescribing. A survey conducted in Poland in 2017 indicated that Polish doctors were aware that electronic prescribing could eliminate the problem of prescription illegibility and incompleteness. However, they expressed doubts about issues such as appointment duration, legal problems or system implementation costs [24]. One survey showed that e-prescribing took 29 seconds more, on average, than issuing paper prescriptions and generated limited time savings in cases of prescriptions required for treatment continuation [25]. Considering the above and patients’ interests, the Ministry of Health addressed a letter to the Superior Council of Doctors that a paper prescription could be issued even in cases excluded by law; and no penalties for issuing paper prescriptions were stipulated [26]. However, users issuing both paper and electronic prescriptions were frequently observed to give up e-prescribing until the system became obligatory [27,28,29].

Our survey revealed that doctors did not accept that e-prescribing was likely to improve their performance. Our study’s respondents were prone to assertive independence and responded that they were not subject to social influences in their work. These results also showed that most respondents held negative opinions about the level and availability of support during the implementation of e-prescribing in their everyday work.

Low scores in the Performance Expectancy section of our survey indicated that doctors are not convinced that the use of e-prescribing in their everyday work will help them achieve better results. The survey indicated that prescription-issuing doctors generally disagreed with the statement that the technology in question would speed up the performance of their professional duties. They also mostly disagreed with the statement that e-prescribing had a positive impact on performance effectiveness.

Our analysis of responses according to respondents’ relative professional experience, indicated that those with a longer-duration professional experience perceived fewer benefits stemming from the use of e-prescribing than those with a shorter-duration professional experience. This may be a sign that long-held practice habits are likely to be a significant barrier to doctors’ acceptance of new technologies. The literature confirms that doctors are often seen to be unwilling to accept changes, if the added values of the new system are not visible [27,30].

Many studies indicate that e-prescribing has the potential to benefit doctors’ effectiveness and to improve the quality of prescriptions. There are, however, reports that e-prescribing takes more time than traditional prescribing [16,25]. A survey conducted in India in 2018 proved that only 45% of local doctors agreed that electronic prescribing was faster and easier than traditional prescribing. In addition, 35.8% of the Indian respondents stated that e-prescribing interrupted their workflow [31]. E-prescribing may only be fully accepted by doctors if they are strongly convinced that the technology has a positive impact on their effectiveness and efficiency [16].

The general result, a score of 3.13 in the five-point Likert scale for this factor, indicated that for most respondents, achieving a workflow when using e-prescribing was not easy. Other research that has focused on users’ acceptance of technologies suggests that a new system will be regarded as useful when those users also see the system as easy to use. Users are not willing to take advantage of new system functionalities if they believe that the system is difficult to operate [14].

Our survey disproved our hypothesis that the user’s age is an important determinant of an individual’s attitude towards the use of new technologies. Our results bore out the stereotype that younger people have more frequent contact with mobile technologies than the older generations and that therefore they perceive them as more useful and easier to use. It therefore seems obvious that it will be easier to encourage doctors from a younger generation to use e-prescribing than their older professional colleagues.

Although e-prescribing related to a patient’s changing therapy is believed to be more complicated (32.7%), using e-prescribing in extending pharmacotherapy was held to be simpler and faster than former methods, by 77.8% of responding doctors in an Indian study [21].

Based on the data we obtained, the medical profession appears to be a relatively assertive group. The average score in the five-point Likert scale for responses to the Social Influence section was 2.3. Considering our Social Influence results in relation to the relative duration of doctors’ professional experience, our study showed that doctors with a longer-duration of professional experience were more likely to be socially influenced to accept e-prescribing than their less-experienced colleagues. For the doctors with more professional experience, the opinions of other people with professional authority, was of greater importance than for their colleagues with shorter-duration work experience. However, our survey results indicated that doctors do not take the opinions of their relatives into consideration in relation to their acceptance or not of e-prescribing. Younger doctors are generally more assertive and independent in making decisions on how they perform their role, and especially, in their decisions connected with the use of new technologies.

A similar survey conducted in South Africa in 2013 reported that social influence on willingness to use e-prescribing by doctors is small [16]. In a 2008 study on new technology acceptance, regarding a new digital picture archiving and communication radiographic system, it was found that there was no interdependency between social influence and the acceptance of the technology [32].

Our survey data analysis concluded that medical professionals did not feel supported by their direct superiors during the implementation of the e-prescribing system. In addition, the survey indicated that Polish doctors are not convinced that the relevant technical and organisational infrastructure required for supervising the implementation of e-prescribing at their healthcare centre did not exist.

A review of the literature confirms that insufficient technical support is one of the major barriers to the implementation of an e-prescribing system [28]. A healthcare centre that implements e-prescribing in their systems must ensure that the relevant technical support services will also be easily accessible to users and that they will be capable of rapid troubleshooting when issues arise. However, studies showed that user manuals and instructions turned out to be unsatisfactory and unhelpful [28]. The provision of sufficient financial resources to operate the e-prescribing system was also found to be an important success factor [33]. In addition, it was confirmed that proper training contributed to safety improvements in the use of new technologies [34].

An Indian survey indicated that around 60% of Indian doctors had experienced technical problems and needed regular support [31]. In turn, a Chinese study confirmed the hypothesis that the greater organisational support that exists at the healthcare centre level, the use of e-prescribing will be more widespread [35].

One implication of our analysis is that acceptance is likely to improve if e-tools for doctors are designed with the experience-levels of medical professionals in mind, and this includes considering the benefits of standardising the e-prescribing software. Further implications arise in relation to the need to appropriately facilitate the introduction and ongoing use of e-prescribing, with such as proper training, financial resources and technical support. Our study is the first survey in Poland of doctors’ levels of acceptance of the new technology to be conducted using the UTAUT model.

A limitation of our study is the small sample size which did not meet the number of respondents recommended for our sample to representative. The results cannot be extrapolated widely without further research. The shortage of respondents increases the margin of error to 8.15%. However, it has been proven that the UTAUT method is well suited to testing the acceptance of using new technologies such as e-prescriptions. This study can be considered as a reliable pilot study that provides the basis for further research with a larger number of respondents.

## 5. Conclusions

Our survey results support the conclusion that how easy and intuitive an e-tool is for users will significantly influence its acceptance. Therefore, we suggest that at the design stage for any health system e-tools, the experience-levels of the end users should be considered, and that clear demonstrations of how easy-to-use the new technology is should be provided. Related to these matters, we have concluded that proper training should include clear and convincing evidence of the added values of using the new technology.

Our study has indicated that no particular benefit is to be gained by the participation of authority figures in the promotion of e-prescribing or other technologies among younger doctors, because this social group is highly assertive.

The provision of relevant technical and financial support during the implementation of new technologies at healthcare centres appears to be a significant factor in encouraging the uptake and continued use of e-prescribing, and it is likely this will also apply to other new technologies. Adequately planned training is also important during the implementation of modern e-services in the healthcare system.

## Figures and Tables

**Table 1:** Analysis of the frequency of a variable: age.

Variable Levels	Frequency	Percent	Frequency	Percent
<30 years	45	31.47	<30 years	31.47
30–40 years	59	41.26	30–40 years	41.26
40–50 years	18	12.59	>40 years	27.27
>50 years	21	14.69		
Total	143	100.00	143	100.00

**Table 2:** Reliability statistics for individual subscales of the UTAUT Questionnaire.

Scale	Cronbach Alpha	Number of Positions
Performance Expectancy	0.96	4
Effort Expectancy	0.88	4
Social Influence	0.57	3
Facilitating Condition	0.88	4

**Table 3:** Regression coefficients—impact of UTAUT factors on e-prescribing frequency.

UTAUT Factor	*Beta*	*p*	95.0% Confidence Interval for *B*	
			Lower Limit	Upper Limit
(Fixed)		0	2.82	3.18
Performance Expectancy	0.21	0.034 *	0.02	0.47
Effort Expectancy	0.23	0.036 *	0.02	0.53
Social Influence	0.16	0.086	−0.03	0.39
Facilitating Conditions	–0.02	0.847	−0.26	0.21

Note: *. *p* < 0.05.

**Table 4:** Relation between age and individual UTAUT factors.

Variable	df	F	*p*
Performance Expectancy	2	2.31	0.103
Effort Expectancy	2	3.28	0.041 *
Social Influence	2	2.98	0.054 *
Facilitating Conditions	2	0.48	0.617
Total	142		
Age ranges: <30 years old, 30–40 years old, >40 years old			

Note: *. *p* < 0.05.

**Table 5:** Impact of professional experience on individual UTAUT factors.

Model	R	R^2^	Adjusted R^2^	SE	Model Adjustment Statistics				DW
					F	df_1_	df_2_	Significance F	
1	0.17	0.03	0.02	1.20	3.96	1	141	0.049	1.92
2	0.15	0.02	0.01	1.11	3.14	1	141	0.078	1.75
3	0.17	0.03	0.02	0.75	4.18	1	141	0.043	1.80
4	0.05	0.00	0.00	1.16	0.33	1	141	0.566	1.78

Note: Model 1= dependent variable: Performance Expectancy; Model 2 = dependent variable: Effort Expectancy; Model 3 = dependent variable: Social Influence; Model 4 = dependent variable: Facilitating Conditions.
